# Proline Tagging for Stress Tolerance in Plants

**DOI:** 10.1155/ijog/9348557

**Published:** 2025-04-02

**Authors:** Naveed Ul Mushtaq, Seerat Saleem, Aadil Rasool, Wasifa Hafiz Shah, Inayatullah Tahir, Chandra Shekhar Seth, Reiaz Ul Rehman

**Affiliations:** ^1^Department of Bioresources, School of Biological Sciences, University of Kashmir 190006, Srinagar, India; ^2^Department of Botany, School of Biological Sciences, University of Kashmir 190006, Srinagar, India; ^3^Department of Botany, University of Delhi 110007, New Delhi, Delhi, India

**Keywords:** abiotic stress, biosynthesis, glutamate, ornithine, proline transporters

## Abstract

In environments with high levels of stress conditions, plants accumulate various metabolic products under stress conditions. Among these products, amino acids have a cardinal role in supporting and maintaining plant developmental processes. The increase in proline content and stress tolerance in plants has been found optimistic, suggesting the importance of proline in mitigating stress through osmotic adjustments. Exogenous application and pretreatment of plants with proline increase growth and development under various stressful conditions, but excessive proline has negative influence on growth. Proline has two biosynthetic routes: glutamate or the ornithine pathway, and whether plants synthesize proline by glutamate or ornithine precursors is still debatable as relatively little is known about it. Plants have the innate machinery to synthesize proline from both pathways, but the switch of a particular pathway under which it can be activated and deactivated depends upon various factors. Therefore, in this review, we elucidate the importance of proline in stress mitigation; the optimal amount of proline required for maximum benefit; levels at which it inhibits the growth, conditions, and factors that regulate proline biosynthesis; and lastly, how we can benefit from all these answers to obtain better stress tolerance in plants.

## 1. Introduction

Due to their sessile nature, plants are prone to environmental variations, such as biotic and abiotic stressors [1]. The stress caused due to salinity is the most injurious abiotic stresses that influence both the quality and quantity of the crops and thus needs to be addressed [2]. Salinization occurs naturally, but it can also be induced by human intervention, such as saline irrigation, seawater intrusion, and fertilizer usage. Most crop plants are severely affected by salty soil because it contains high levels of soluble salt [3, 4]. The amount of salt-affected soils worldwide is not precisely estimated, and different data sources give differing information in this regard. However, reports have also indicated 932.2 million, 952.2 million, and 1000 million hectares of salinity-affected soils globally [2, 5]. Approximately, more than 6% of irrigated and almost 20% of the world's cultivable land is affected by salinity. This rate is expanding at a rate of 1%–2% per year, and this will result in more than half of nonproductive land by the year 2050 [6]. In light of the above facts, we face a growing challenge to ensuring global food security. In such conditions, it is even more imperative to find approaches for improving the productivity of soils with salinity, and further, it is also cardinal to screen/develop varieties that are efficient and tolerant to salty soil. Thus, in order to explore alternative methods for improving crop tolerance and accomplishing sustainable development objectives, more research and studies in the field of crop science are required.

**Figure 1:**
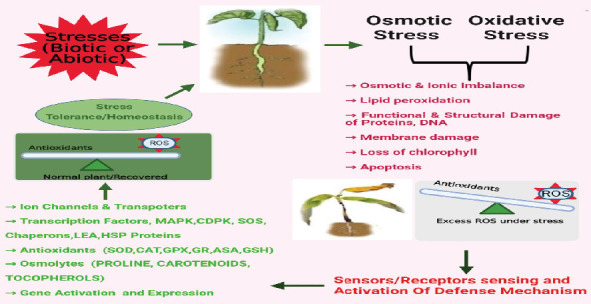
Mechanism of plants to withstand stress. ROS, reactive oxygen species; MAPK, mitogen-activated protein kinase; CDPK, calcium-dependent protein kinases; SOS, salt overly sensitive; SOD, superoxide dismutases; CAT, catalase; GPX, glutathione peroxidase; GR, glutathione reductase; ASA, ascorbate; GSH, glutathione.

After the exposure of plants to saline conditions, the salinity is perceived by sodium ions (Na^+^) and stress-related signals [7]. Osmotic signals are identified by mechanosensors in the cytoskeleton, stretch-dependent/activated ion channels, redox-mediated systems, protein kinases, and wall-related kinases [8]. Further, the receptors on the membrane may be associated with the sensing of extracellular salt, whereas intracellular sodium may be sensed by sodium-sensitive enzymes and membrane proteins in the cell cytosol. Na^+^/H^+^ antiporter SOS1 is one of the Na sensors that has a role in Na^+^ efflux in plant cells. Many studies also revealed that SOS5 is also responsible for Na^+^ [9, 10]. On the other hand, the stress-related signals are activated by abscisic acid (ABA), which upregulates the *AtNHX1* gene and leads to amassing of Na^+^ in the vacuole [11, 12]. Similarly, osmotic stress triggers increase in [Ca^2+^]cyt which activates phosphatase 2B calcineurin and leads to activation of ENA1 encoding the P-type ATPase which has a role in sodium efflux [13, 14]. Apart from this, under a saline environment, it is crucial for plants to perform osmotic regulations by accumulating solutes such as proline, betaine, and polyols. These solutes had a vital role in reducing the adverse consequences of salinity stress (Figure 1) [15, 16].

Reactive oxygen species (ROS) and hydrogen peroxide (H_2_O_2_) also function as signaling molecules that set off specific subsequent events [17]. In response to H_2_O_2_, two mitogen-activated protein kinases (MAPKs), MPK3 and MPK6, are required for signal transduction in *Arabidopsis thaliana* [18, 19]. In plants, OXI1 kinase serves as ROS sensor and activates MPK3 and MPK6 by detecting ROS [20], whereas *OMTK1* has a MAPK scaffolding role and triggers H_2_O_2_-induced cell death in plants [21]. H_2_O_2_, whether directly or indirectly, stimulates antioxidant enzymes and genes like APX and CAT, resulting in ROS detoxification [22]. Additionally, proline plays a critical function in signaling, scavenging, cell reprogramming, programmed cell death, aging, and hypersensitive reactions [23]. Besides, proline acts as an osmolyte, stabilizing various cellular structures, functions in various pathways, maintains a redox state, and functions as a metal chelator, signaling molecule, and antioxidant defence [24, 25]. Proline endogenously accumulates under stressful conditions, and the quantity of proline accumulation differs within and among species and depends on the degree of stress [15, 25].

Proline is a standard amino acid possessing a cyclic structure and the nonpolar side chains that aid in cellular regulation as it subsidizes in balancing cellular structures (like biomembranes, lipids, enzymes, and amino acids), acts as antioxidant, and thus maintains cellular redox potential during stress [26–28]. It plays a significant role in plant enhancements, flowering, pollen development, embryo development, and leaf expansion. With stress, an increase in proline quantity typically occurs within the cytosol, resulting in an adjustment in osmotic balance [29, 30]. It has noteworthy character in stress conditions as it acts as a metal chelator, ROS scavenger, and signaling molecule. Various studies demonstrated that mild applications of proline may offer protection and help alleviate the harmful impacts of ROS, salinity, drought stress, heavy metals, and cold conditions [31, 32]. Proline hyper buildup enhanced heavy metal tolerance in plants and algae [33, 34] and reduced apoptosis in fungi *Colletotrichum trifolii* [35]. Similarly, it has been observed that *Arabidopsis thaliana* mutants with impaired proline synthesis exhibited an increased vulnerability to salt stress [36]. Due to the advantageous attributes exhibited by proline, it is deemed valuable to examine its biosynthetic pathways, elicitation methods, inhibitors, chelators, mitigation strategies, and its relation with other metabolic cycles. This comprehensive review is aimed at answering these questions, hence facilitating its effective utilization as a strategy for enhancing salt tolerance and agricultural productivity.

## 2. Proline Biosynthesis and Degradation in Plants

**Figure 2:**
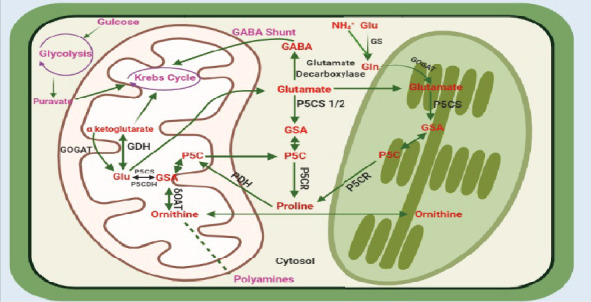
Overview of the proline biosynthesis pathway and the different enzymes involved. GDH, glutamate dehydrogenase; P5CS, pyrroline-5-carboxylate synthase; P5CR, pyrroline-5-carboxylate reductase; PDH, proline dehydrogenase; GS, glutamine synthetase; GOGAT, glutamate synthase; P5C, pyrroline 5-carboxylate; GSA, glutamic semialdehyde; Glu, glutamate; OAT, ornithine aminotransferase.

Proline synthesis in higher plants can be achieved through two distinct pathways, depending on the availability of glutamate (Glu) and ornithine (Orn), as well as the specific conditions within the plants. It is still unclear which substrate is preferentially used by proline biosynthesis, even though the different genes and enzymes in the proline pathway have been well characterised [37]. The available information revealed that the preferred pathway of proline biosynthesis is influenced by the developmental stage of the plant, the species under study, the concentration, and the type of stress involved [30, 38, 39]. The initial report on the biosynthesis of proline in *E. coli* was reported in 1952 [40]. The initial identification of the genes needed for proline synthesis in plants was found to be present in the cytosol. However, further research revealed a relocation of these genes to the chloroplasts [41]. The core proline metabolic pathway includes a pair of enzymes that catalyse the biosynthesis of proline from Glu, occurring in either the cytoplasm or chloroplasts [16, 42]. Additionally, two enzymes are responsible for the catabolism of proline, converting it back into Glu within the mitochondria. Additionally, an alternate pathway for the synthesis of proline, including the utilization of Orn as a substrate, is catalysed by ornithine aminotransferase (OAT) [43] (Figure 2). It is hypothesised that in unfavorable circumstances, there is an upregulation of proline synthesis from Glu, along with a downregulation of proline catabolism, which is thought to play a role in the regulation of proline levels and contribute to stress alleviation. The synthesis of proline in plants takes place in several cellular compartments and is influenced by environmental cues. The synthesis of proline occurs through a cascade of enzymatic events using *Δ*1-pyrroline-5-carboxylate (P5C) as an intermediate, which is derived from either Glu or glutamic acid. The aforementioned procedure encompasses a series of two successive reductions facilitated by the enzymes *Δ*1-pyrroline-5-carboxylate synthase (P5CS) and *Δ*1-pyrroline-5-carboxylate reductase (P5CR). It is worth mentioning that P5CS employs NADPH as a cofactor, whereas P5CR relies on NADH [32, 44, 45]. The encoding of P5CS in the majority of plants involves two genes, while P5CR is encoded by a single gene [41]. Moreover, the synthesis of proline can be achieved by the conversion of Orn, which is assisted by the enzymatic action of *δ*OAT (ornithine *δ*-aminotransferase). Nevertheless, it is important to acknowledge that *δ*OAT seems to have a greater impact on nitrogen recycling rather than serving as the main contributor to proline synthesis [43]. Overexpression of *Arabidopsis δOAT* gene in tobacco and rice had amplified proline content and increased stress tolerance [46, 47]. The use of “gabaculine” as an inhibitor of OAT in radish (*Raphanus sativus*) under salt stress revealed the role of the Orn pathway in proline biosynthesis [48]. Improved activities of P5CS, OAT, and proline content were found in *Arabidopsis thaliana* under salt-stressed conditions [28]. In switchgrass, *PvP5CS2* had a role in reproductive growth and the overexpression of *PvP5CS1* and *PvP5CS2* resulted in the recovery of plants under high salt treatment suggesting the significance of proline in suppressing salt-provoked physiological harms [42]. During osmotic stress, Glu pathway accounts for the increase in primary proline. In another study, the enhancement of salt tolerance in switchgrass was achieved by the overexpression of *P5CS* genes derived from *Lolium perenne* and *Puccinellia chinampoensis*. The results of the study indicated a reduction in electrolyte leakage and levels of ROS [49]. The degradation of proline is catalyzed by successive activity of two mitochondrial enzymes, proline dehydrogenase (PDH) and *Δ*^1^-pyrroline-5-carboxylate dehydrogenase (P5CDH). *PDH* is encoded by two genes and *P5CDH* by one gene as identified in *Arabidopsis* and tobacco (*Nicotiana tabacum*) [50, 51]. FAD is a cofactor of PDH, whereas NAD^+^ acts as cofactor for P5CDH uses cofactor.

In mitochondria, the degradation of proline leads to the formation of arginine from Orn via the urea cycle. Arginine is converted into agmantine by arginine decarboxylase, or it can lead to the production of nitric oxide (NO). Further agmantine is the precursor of putrescine and polyamines (PAs) which have a role in environmental stresses like osmotic stress [52], drought [53], heavy metals [54], and UV radiation. PAs are also concerned with the procurement of tolerance to hypoxia, salt stress, hyperosmosis, and atmospheric pollutants [55, 56].

### 2.1. Regulation of Proline Biosynthesis

The regulation of proline is governed by MAPK cascades [28]. The receptors of this cascade are modulated with different stimuli. Under stressful conditions, MAPK is a key signaling route for the synthesis of L-proline. There are three components of the MAPK signaling pathway, that is, MAP kinase kinase kinases, mitogen-activated protein kinase kinases (MAP2Ks), and MAP kinases [57]. Serine/threonine kinase phosphorylation occurs under stimulation, and the MAPK receptor undergoes the phosphorylation and turns active; MAPKs are phosphorylated by MAP2Ks at the conserved T-X-Y motif on the threonine and tyrosine residues [58]. As a result of stress, MAP kinase activates and synthesizes L-proline as well as phosphorylates various substrates, kinases, and transcriptional factors. Additionally, recent research demonstrates that ABA signaling and phosphate homeostasis are involved in the regulation of proline biosynthesis, and the regulation of phosphate homeostasis is done via Phosphate Starvation Response 1 (PHR1) and PHR1-LIKE 1 mediated transcriptional activation. Besides ABA, P5CS genes and pro-biosynthesis are also regulated by calcium, light, and lipid signals [59]. The role of ABA in the upregulation of stress-induced genes has been extensively studied. However, previous studies have presented conflicting data regarding the regulation of either ABA-dependent or ABA-independent mechanisms of proline synthesis. Research on ABA-deficient mutants and the application of exogenous ABA has provided insights into the regulation of proline accumulation during stress [60, 61]. It has been observed that ABA plays a partial role in this process. However, it is important to note that the application of ABA without stress is not enough to accumulate significant amounts of proline [62]. In a recent study, researchers conducted a comparative analysis of *P5CS1* expression in relation to identified ABA-induced genes and the findings revealed that the regulation of *P5CS1* is largely independent of ABA [63]. The current understanding of the signal transduction pathway regulating *P5CS1* remains limited, and it is believed that the involvement of phospholipase C and calcium signaling may have a potential role in the upregulation of *P5CS1* expression in *Arabidopsis* under salt stress [64, 65].

## 3. Switching Between Glu and Orn Pathways Under Varying Conditions

Whether plants synthesize proline by Glu or Orn precursors is very important which is desirable as there is scarcity of information about it. Plants have the innate machinery to synthesize proline from both pathways, but the switch between these depends on the environmental conditions under which they can be activated or deactivated. The study on salt stress and proline metabolism in various plants revealed improvements in proline content in leaves which further improved with salt stress in a dose-dependent manner [66–68]. Similarly, the improved activity of enzyme P5CS was found initially which decreased with increasing NaCl concentration. The activities of *δ*OAT and PDH were amplified and decreased, respectively, in response to elevated NaCl concentration and treatment times. The activities of P5CS increased at low concentration of salt while as the *δ*OAT activity increased at elevated salt concentration. However, the activity of proline (PDH) decreased in response to elevated salt and is time dependent. Thus, the Glu and Orn pathways are activated by differential salt concentrations. However, it is noteworthy to mention that most plants in nature are subjected to lower salt concentrations; thus, Glu pathway is responsible for proline synthesis, while the Orn pathway is activated under high application of salt [24]. The levels of P5CR were found to be significantly higher in detached *Oryza sativa* leaves treated with copper sulfate (CuSO_4_) compared to those treated with water. This observation suggests that the excess CuSO_4_ exposure leads to the conversion of glutamic acid to proline in rice leaves. The findings of this study were further confirmed by the observation of reduced levels of glutamic acid in detached rice leaves subjected to CuSO_4_-induced stress [69]. Further, OAT activity also increased significantly in CuSO_4_-treated leaves. Comparable results were obtained in wheat when subjected to cold stress, it also resulted in increased OAT activity and proline content [70].

The accessibility of nitrogen also has an effect on proline buildup in a number of plants. Tobacco leaves grown on a medium having high amounts of ammonium nitrate (NH_4_NO_3_) show increased proline content. In roots and leaves of *Phaseolus vulgaris*, *δ*OAT activity was optimistically linked with proline applications over a wide range of NH_4_NO_3_, while P5CS activity was adversely controlled [71].

Roosens et al. [72] concluded that the Orn path has an important function in young, but not in adult, *Arabidopsis thaliana* plantlets. Salt stress in *Brassica napus* induced proline accumulation resulting from the reciprocal action of activated synthesis and repressed proline degradation. Additionally, the Orn pathway seems to add to the proline accumulation under long-lasting osmotic stress [73]. However, in *Oryza sativa* leaves, it was found that it contributes small to proline amassing under water stress circumstances [74]. This was unlike for P5CS and made known that under NaCl stress circumstances, the Orn pathway is not a major pathway. In yet another report in *Helianthus tuberosus*, Orn was also the contributor for proline synthesis, and Glu is the main precursor [75]. The clone of OAT was isolated from *Arabidopsis*. The researchers aimed to investigate the expression of this gene under salt stress conditions and found opposite findings. The observed variations in OAT activity can potentially be attributed to the diversity of species examined in the studies, which encompassed a range of conditions, ages, and developmental stages. Thus, both Glu and Orn pathways come into light as important under abiotic stress in plants [76].

### 3.1. Crosstalk in Proline Biosynthetic Pathway Elevates Proline for Cellular Homeostasis

The accumulation of proline is a natural response to normal as well as stress conditions, but the overproduction of proline may cause an imbalance in cellular homeostasis. P5CS is an enzyme that acts as a limiting factor in the production and control of proline accumulation. In *Arabidopsis thaliana*, two genes encoded *P5CS*, that is, *P5CS1* and *P5CS2*. The latter is regarded to be a housekeeping gene because it is expressed throughout the plant, whereas *P5CS1* reacts to stress situations. In *A. thaliana*, dehydration, high salt, and other stresses produce isoform1; however, isoform2 is essential for embryo and seedling growth. The preservation of tertiary structure and protein–protein interaction is aided by the leucine zipper sequence of this enzyme [77]. The divergent transcription patterns of *P5CS1* and *P5CS2* indicate that they have diverse functions in both developmental processes and stress responses. The observed genetic underpinnings of the naturally occurring variation in proline concentration in *Arabidopsis thaliana* seem to be linked to the specific alternative splicing pattern shown by *P5CS1*. Epigenetic regulation is also evidently implicated, since changes in DNA methylation levels seem to promote proline accumulation through the upregulation of *P5CS* and *OAT* [78]. Proline, being an amino acid that responds to stress, has the ability to accumulate under unfavourable environmental conditions and serves as a signal for the transmission of stress memory to subsequent generations in a stable manner [25]. Also, the transcription of *P5CS1* in the vegetative tissues of the plant can be induced by the exogenous application of several agents such as ABA, PAs, proline, zinc, and selenium (Se). A variety of factors influence the activity of *P5CS*. Several studies have found increased expression of *P5CS* under salt stress. Kubala et al. [79] revealed increased expression of *P5CS* in *Brassica napus*, [80] in tomato, [75] in *Jerusalem artichoke*, and [81] in *Arabidopsis* under salt stress. Similar observations were reported in various plants like carrot, cactus pear, sugarcane, rape seed, and mustard [10, 79, 82–84].

*P5CS1* expression increases in various plant species under stress, but *ProDH* expression decreases. *PRODH1* and *PRODH2* are isoforms of PDH, the rate-limiting enzyme in two mitochondrial processes that convert proline to Glu. Proline oxidation produces 30 adenosine triphosphate (ATP) molecules and Glu, which then enters the tricarboxylic acid cycle (TCA) cycle as *α*-ketoglutarate. In addition to giving reducing power to the mitochondria or creating ATP, PDH supplies nitrogen from proline [85]. This enzyme works with P5CDH and GDH (glutamate dehydrogenase) and supplies carbon to the TCA cycle. PDH responds to environmental stress, and its activity increases to break down extra proline after stress levels are relieved [16]. PDH activity is influenced by salt, drought, low temperatures, heavy metals, and other abiotic and biotic stresses. Decreased PDH activity under various stressful conditions has been observed in many plants, including *Mangifera indica* L., *Brassica napus*, *Thellungiella salsuginea*, *Nitraria tangutorum* Bobr., *Zea mays* L., *Cucumis sativus*., *Setaria italica* L., and *Pisum sativum* [79, 86–92].

OAT, also known as *δ*OAT, is a pyridoxal phosphate (PLP)-dependent enzyme. In plants, proline is primarily synthesized via the Glu pathway under osmotic stress and nitrogen limitation, while the Orn pathway may become active under conditions of high nitrogen input [93]. The activity of OAT is influenced by various factors such as salt, drought, and biotic stresses [94, 95]. Research has shown that overexpressing the *Arabidopsis δOAT* in tobacco and rice leads to increased proline levels and enhanced tolerance to biotic and abiotic stresses [96]. Recent studies have found that OAT activity increases three-fold under osmotic stress, such as in NaCl-treated wheat, soybean, and rice [46, 63, 97–99]. However, decreased proline content was observed when OAT was inhibited by gabaculine in radish cotyledons and detached rice leaves [100, 101]. Additionally, studies in rice, cashew, and European searocket revealed that the Orn pathway is activated at higher stress levels, whereas the Glu pathway is more active at lower stress levels [102–104].

A plant's autotrophy relies heavily on glutamine synthetase (GS), an enzyme that converts ammonia into Glu to generate glutamine and hydrolyzes ATP at the same time, thus having a vital function in assimilation and/or reassimilation of ammonia [105]. The regulation of GS in plants is extremely significant since it is a central enzyme in nitrogen metabolism, nitrogen use efficiency, and plant development and catalyzes the fixation of ammonium to the *δ*-carboxyl group of Glu to form glutamine [106]. Glutamine and 2-oxoglutarate are converted to two molecules of Glu by glutamate synthase (GOGAT); thus, this enzyme provides the Glu for ammonium assimilation. As a result of the GS–GOGAT cycle, Glu is produced, which can be converted to other amino acids including proline by aminotransferases or transaminases [107]. The activity of this enzyme is influenced by various factors like salt, metal stress, cold, drought, and biotic factors. The GS activity was enhanced by the salt stress in cashew, rice, tomato, and potato [108–111], under excess ammonia in spinach, tomato, lettuce, and pea [112].

## 4. Proline Biosynthetic Pathway and Its Relation With Other Pathways

The process of proline biosynthesis utilizes NADPH as a reducing agent and is known to be associated with the pentose phosphate pathway (PPP), which serves as a means of transporting the reducing agents and maintaining the redox balance inside the chloroplast. The PPP and glycolysis are responsible for generating NADPH, which is essential for various biosynthetic processes [113]. When plants are exposed to stress conditions such as drought or salinity, they often accumulate proline as a protective mechanism. In the biosynthesis pathway of proline, NADPH is consumed, and the regeneration of NADPH can be supported by the PPP and glycolysis. Further, ribulose-5-phosphate formed from the pentose phosphate cycle enters the Calvin cycle (can enter through phosphoribulokinase or can be metabolized to form glyceraldehyde-3-phosphate) with a chain of reactions in nonoxidative phase of PPP. The synthesis of ribulose-5-phosphate is accompanied by the generation of two molecules of NADPH and one molecule of carbon dioxide (CO_2_) [114, 115]. The NADPH generated is not only used in the biosynthesis pathway of proline but also for CO_2_ assimilation. These mechanisms may have advantageous effects during circumstances of stress, whereby there is a restricted availability of CO_2_ as a result of stomata closure. The NADPH is also used to regenerate reduced glutathione, by NADPH-dependent glutathione reductase (GR) in the ascorbate–glutathione pathway [116].

This enzyme converts proline to P5C on the matrix side of the inner mitochondrial membrane and moves electrons to the mitochondrial electron transport chain (ETC) [117]. Proline's oxidation yields 30 ATP molecules and Glu, which subsequently enters the TCA cycle as *α*-ketoglutarate. PDH also uses proline to produce nitrogen in addition to ATP [85]. Additionally, this enzyme supplies carbon to the TCA cycle in combination with P5CDH and GDH. In the synthesis of proline, the TCA cycle produces 2-oxoglutarate, which can serve as a source of nitrogen for the synthesis of other amino acids, such as Glu and glutamine. The nitrogen atoms in these amino acids can be recycled and incorporated into proline through the P5CS-mediated pathway when plants need to accumulate proline as a response to stress or other physiological conditions. The GDH enzyme in higher plants is responsible for facilitating the reversible amination process of 2-oxoglutarate, resulting in the formation of Glu [118]. This enzymatic reaction utilizes ammonium as a substrate. The primary function of GDH is to provide fuel to the TCA by supplying 2OG when carbon availability becomes restricted. In conjunction with GOGAT, GDH regulates the maintenance of Glu homeostasis. Therefore, it can be inferred that GDH plays a crucial role in maintaining Glu homeostasis and serves as a key component at the intersection of carbon and nitrogen absorption pathways [119]. Theoretically, this enzyme has the capacity to either incorporate or release ammonium. However, a significant data has indicated that the majority of ammonium used by higher plants is absorbed by the GS–GOGAT route [118]. However, several studies suggest that GDH may function in the process of ammonium absorption, particularly in situations when stress circumstances promote the buildup of ammonium. On the other hand, some researchers contend that under normal growth settings, GDH acts by deaminating Glu to produce ammonium [119, 120]. Thus, under abundant ammonia supply or adverse environmental conditions, the enzyme GDH plays an important role. The deaminating role of this enzyme contributes to the safe operation of the TCA cycle, especially when the amount of carbon is limited [121, 122]. So, it can be concluded that proline metabolism is also linked to Kreb's cycle or citric acid cycle and ammonia cycle. The GABA shunt at the side of proline metabolism is also linked to other pathways. The GABA shunt is a metabolic pathway in plants that entails the conversion of GABA to succinic semialdehyde, which is then converted to succinate and enters the TCA cycle [74]. The activation of GABA shunt results in the production of succinate, which plays a critical role in stress tolerance and adaptation in plants by providing energy and carbon skeletons for biosynthesis and stress-responsive compounds [123]. The relationship between PA biosynthesis and the proline pathway is established through the shared utilization of Orn as a common precursor for both pathways. The utilization of Orn as a substrate for proline synthesis has been extensively studied [124]. The most prevalent PAs found in plants include putrescine, spermine, and spermidine. Putrescine is a compound that is synthesized through the process of decarboxylation by an enzyme called Orn decarboxylase. Alternatively, putrescine can also be indirectly synthesized through the decarboxylation of arginine, which is facilitated by an enzyme known as arginine decarboxylase. This indirect pathway involves the intermediate formation of agmatine before ultimately leading to the production of putrescine. Furthermore, it is worth noting that the synthesis of other PAs occurs in a sequential manner following the production of putrescine [125]. The synergistic action of proline and PAs has been found to play a crucial role in safeguarding plant cells by preserving cellular homeostasis during periods of stress [126]. The research pertaining to the coordination of proline with fatty acid synthesis unveiled a connection between proline metabolism, lipid metabolism, and the redox metabolism of mitochondria and chloroplasts. The study not only concluded that proline metabolism is influenced by cellular redox conditions but also found a dual role shared by proline and lipid metabolism. In this way, they help the plant by balancing the cellular redox state and by producing the lipids required to form cuticles [127].

## 5. Is More Proline Better for Stress Mitigation in Plants

Under ordinary situations, proline encompasses 5% or less of the total free amino acids [128]. The impact of various stresses on the changes observed in the amino acid pool indicated a significant increase of up to 80% in the amino acid pool under stress conditions. Notably, the extent of this boost varied across different plant species, with some exhibiting levels that were more than 100 times higher than the control group. Extra exogenous proline has an adverse outcome on plants in lieu of a defensive role under stress situations [50, 129]. The extreme applications of exogenous proline hamper the development while lower concentrations increase the growth, as observed in *Arabidopsis* explants [130]. It was reported in *Arabidopsis* that higher concentrations of proline restrain the P5CS and hinder the organogenesis [131, 132]. Similarly in tomatoes, a higher concentration of proline showed a depressing effect on growth and imbalance in the inorganic ions [133]. The minimum and maximum concentrations of proline are nearly 93.2% and 98.6%, correspondingly [134].

Excessive foliar application of proline has been found to have a detrimental impact on plant metabolism as it shows high degrees of toxicity and cell death, which may also be due to the breakdown of proline into lethal intermediates [129]. P5C cycle boosts under the application of exogenous proline resulting in the transfer of electron to reactive ROS [135]. It was demonstrated in rice that proline at low concentration (20-30 mM) diminished the unfavourable effects of salinity but the higher concentrations (40-50 mM) of proline resulted in toxic effects and reduced growth [136]. Similarly, lower applications of proline (5 mM) were more efficient in *Vicia faba* and eased harmful effects in ground nuts [137]. It was also revealed that pretreatment of rice seeds with proline (1 mM) reduces the negative effects of NaCl stress, whereas proline at high quantity (10 mM) was detrimental [138]. Additionally, the exogenous application of 10 mM proline stimulates growth and boosts the performance of antioxidant enzymes [139].

## 6. Engineering Proline Metabolism for Stress Tolerance

Targeted expression of genes is the key to achieve advantageous metabolic result in plants [140]. The involvement of proline in stress mitigation and tolerance by means of a transgenics approach has been the core of researchers for years [141–143]. The introduction of the *P5CS* gene from *Vigna aconitifolia* into *Nicotiana tabacum* led to a significant increase in proline synthesis, ranging from 10 to 18 times higher than the control [144, 145]. Similarly, elevated resistance to salt stress and increased amounts of proline were conferred in genetically engineered *Oryza sativa* by the expression of *P5CS* from *Vigna aconitifolia* [146]. The rice *P5CS2* knockout resulted in reduced salt tolerance, and the overexpression of *P5CS2* led to increased proline accumulation and enhanced stress tolerance [147]. In another study, the overexpression of proline biosynthesis genes in *Nicotiana tabacum* resulted in an increase in proline intensity which leads to a reduction in free radicals [148]. In the same way, when *StP5CS* gene from *Solanum torvum* was overexpressed in modified soybeans, it resulted in expanded salt resilience and other growth parameters [80]. The transgenic *Solanum tuberosum* expressing the *P5CS* gene sourced from *Arabidopsis* demonstrated enhanced tolerance to 100 mM NaCl-induced stress, improvement in proline content, osmotic potential, biomass production, and bloom advancement [149]. Similarly, higher levels of proline and enhanced tolerance to cadmium stress were found in transgenic *Medicago truncatula*, expressing the *Vigna aconitifolia VaP5CS* [150]. The introduction of *P5CS* in wheat aided in the amplified assembly of proline and easiness to 200 mM salt stress. The increased proline in transgenics also resulted in decreased lipid peroxidation under other stresses [151, 152]. In *Nicotiana tabacum*, improvement in leaves and roots under salt and drought stress was found when *P5CS* was overexpressed [153, 154]. The overexpression of the *P5CS* gene from *V.aconitifolia* into *Citrus sinensis* resulted in better osmotic adjustments and photosynthetic rate [155]. Inhibition of proline breakdown resulted in high salinity tolerance of about 600 mM NaCl in transgenic plants in comparison with wild type [156]. In *Oryza sativa*, overexpression of *OsProDH* decreased proline content, while mutation of *OsProDH* increased proline content compared with that of normal cultivar [157]. In another study related to hybrid proline-rich proteins (HyPRPs), it was found that these proteins can act as both negative and positive regulators under stress. It was revealed that HyPRPs are key proteins for crop improvement and stress tolerance [143]. Several transcription factors including WRKY, bZIP, MYB, NAC, and AP2/ERF are correlated with the expression of genes that encode proline metabolism enzymes e.g. SNAC2 enhanced expression of *OAT* when cloned in rice [41]. Tables 1 and 2 show salt-related genes introduced into crops and enzymes involved in the network of proline biosynthesis and degradation, whereas Tables 3 and 4 show pathway elicitors and inhibitors, respectively.

## 7. Proline Transport in Plants

The transport of proline within the cell and among the cells is vital for retaining the balance; however, the transport of proline within a cell has a significance, as it boosts stress capabilities [179]. The transportation of amino acids depends both on internal and external environmental signals [162, 180]. Further, the stressful environment also impacts the transport of proline. The transporters which are responsible for the transport of proline in *Arabidopsis* are grouped into two superfamilies, that is, the PA, choline, and amino acid transport superfamily and the amino acid transporter family (ATF) superfamily [181, 182]. Additionally, the ATF is divided into amino acid permeases, histidine transporters, lysine, auxin transporters, and L-proline transporters (ProTs) [30]. Under water stress situations, two ProTs ProT1 and ProT2 were identified. These transporters have a role in the transport of proline as well as nitrogen in *A. thaliana*. Further, AAP6 is also found in *A. thaliana* [182, 183]. The transport of proline across the plasma membrane is facilitated by two families of transporters, that is, the amino acid/auxin permease (AAAP) family and the amino acid PA choline family. These ProTs are present within the plasma membrane and are responsible for facilitating long-distance intercellular transport [30, 184]. In phloem and xylem cells, the effect of GS in directing the proline transport was also revealed [185]. In mestome sheath and lateral root cap cells of barley a gene *HvProT2*, coding for the transporter of proline is found [186]. RNA in situ hybridization showed that LeProTs ProT was found in pollens of tomato [187]. Proline uptake into mitochondria is intervened by proline uniporter and proline/Glu antiport system [188]. There are not yet any genes characterized for intracellular transporters in plants, but Glu /proline antiporters have already been identified in mammals [189]. A comprehensive investigation into the mechanisms of metabolism and transport could potentially enhance the understanding related to the significance and regulation of proline homeostasis in plants.

## 8. Mitigation of Salt Stress by Proline

Proline has been employed as a mitigating agent to alleviate stress in many plant species [15]. A significant elevation in proline content was observed when exogenous Se was applied as a mitigating agent in *Setaria italica* L. and *Panicum miliaceum* L. [170, 190]. Similarly, the damages of stress were reduced when the seeds of rice were pretreated with different concentrations of proline. It was found that pretreatment with proline at 1 mM concentration was effective and encouraged cellular activities, whereas 10 mM proline was found to be unproductive in increasing plant growth [138].

Likewise, the low doses of proline used exogenously resulted in reduction of damages caused by salinity stress in various plants [30, 191]. Additionally, proline not only improved the fresh weight of various plants but also resulted in a reduction of peroxidative damage to membranes [137, 192, 193]. Further, it is suggested that proline defends plants from stress, possibly by relaxing the biomembranes, proteins, enzymes, and mitochondrial ETC II [194]. Exogenous proline application was also found to significantly amplify the water potential in leaves during stress conditions [195, 196]. It is also suggested that the buildup of proline in *Opuntia streptacantha* under salt stress may be responsible for intracellular osmotic regulation as well as the role in turgidity and shielding of photosynthetic action in *Opuntia streptacantha* [10].

It was also found that elevated proline in plants grown under saline stress treatment adds to tolerance [197]. Similarly, it was also found the accumulation of proline and spermine molecules responsible for salt stress tolerance [198]. It was demonstrated that the interaction between Si and proline has a significant role in raising tolerance to NaCl and Cd^2+^ stresses in *Phaseolus vulgaris* [199]. Foliar use of proline in heavy metal stress not only reduces the depressing effect of metals but also elicits protective machinery in plants [200, 201]. Proline encourages the development of phytochelatins that later chelate heavy metals and diminish the toxicity of stress [30]. It was stated that pretreatment of proline in Cd^2+^-stressed *Solanum nigrum* diminishes the ROS intensity and defends the plasma membrane, thus ensuring the regeneration of shoots [73]. Similarly, a link between amplified proline concentration and elevated biomass was reported in *Arabidopsis* and rice under stress [96, 202]. Reduction in chlorophyll a and carotenoid contents was observed in the Aloe vera plant when treated with salt, whereas pretreatment of the same plant with proline improves such inhibitory effects [203]. In another similar study, pretreatment with proline improved leaf chlorophyll contents under environmental stress [204]. The application of exogenous proline in *Phoenix dactylifera* L. has been observed to reduce the concentration of cadmium in both the roots and leaves. This behaviour could potentially be attributed to the inhibitory effect exerted by proline on the availability of Cd for uptake. The study's findings indicated that proline had an impact on the uptake of Cd or its complexation in roots. In addition, the application of proline during irrigation has been found to potentially decrease the availability of Cd for root uptake through metal-chelation mechanisms in the soil [205].

Optimum temperature is required by every organism and any deviation can cause serious unrest in plants which may disrupt metabolism, protein content, and enzymes. Exogenous application of proline adds to plant growth and crop productivity under chilling stress conditions [206]. Growth, development, and proline synthesis were found to be reduced in *Jatropha curcas* under low nitrogen supply; however, the enhancement in the gene expression of *JcP5CS* and high enzyme activity was found under drought stress, ultimately resulting in increased proline content [207]. Proline not only increases the antioxidant enzyme machinery but also the other enzymes and enzymes of their own biosynthesis. The activity of nitrogenase in drought-stressed soybean nodules was improved when proline was applied exogenously [208].

Kubala et al. [79] demonstrated an enhanced germination rate when osmoprimed as the increase in proline through priming and postpriming germination was linked with upregulation of *P5CSA*, downregulation of *PDH*, and amassing of H_2_O_2_. The *P5CSA* was consistent with the increase in *P5CS* activity. In spite of the favourable results of proline application, it also has toxic properties when hyperaccumulated or given at higher concentrations.

## 9. Conclusion

It is realised from the outlook of this review that proline is essential imino acid for stress tolerance in plants. Application of proline primes the defence and protects the plant from damage, but more exogenous proline usually does not have beneficial effect to plant. Pretreatment of proline at low concentrations is advantageous, whereas at high concentrations, it is ineffective in increasing plant growth. The switch of the proline pathway seems to be dependent on various external and internal factors like the age of plant, concentration of stress, and availability of essential nutrients like nitrogen. However, the detailed proline synthesis, signaling transduction pathway, and the crosstalk are still largely unknown, and further works are needed to investigate the interaction between proline and these responsive molecules. The transport of proline within the cell and among the cells is vital for retaining the balance. However, the transport of proline within a cell has significant part in boosting stress capabilities. Targeted expression of enzymes and transporters to attain desirable metabolic effect is having scope. The transgenic approach and the role of proline in stress tolerance have been the centre of plant biotechnology and stress biology for many years. Further, the gene transfer method can be considered for the accumulation of this “natural” osmolyte to raise tolerance to stress pressure. Thus, proline tagging and other genetic work on proline overcreation in agriculturally vigorous crops might increase their total environmental tolerance and thereby improve productivity.

## Figures and Tables

**Table 1:** Proline-related genes expressed in crop plants.

**Associated genes/species**	**Promoter**	**Crop**	**Result**	**References**
*P5CS* (*V. aconitifolia)*	Act1	Rice	Better development of shoots and roots	[158]
*P5CS (V. aconitifolia)*	AIPC	Wheat	Accumulation of proline for improved tolerance to water deficit	[152]
*P5CS (Pennisetum glaucum)*	Ubiquitin	Tobacco	Higher chlorophyll, relative water, and proline content, and lower malondialdehyde (MDA)	[154]
*ZFP252* (*Oryza sativa)*	35S	Rice	Increase the amount of proline, drought, and salinity tolerance	[159]
*P5CS* (*Vigna aconitifolia)*	35S	Pigeonpea	More proline than control plants and enhances root growth	[160]
*OAT* (*Arabidopsis)*	35S	Tobacco	More proline showed higher biomass and a higher germination rate	[96]
*P5CS* (*Vigna aconitifolia*)	35S	Tobacco	Overproduction of proline also enhanced root biomass and flower development	[161]

**Table 2:** Enzymes involved in the network of proline biosynthesis and degradation pathway in plants.

**Enzymes of proline biosynthetic pathway**	**Localization**	**Characterization method**	**References**
Pyroline-5-carboxylate synthetase (P5CS)	Chloroplastic or cytosolic	Southern analysis	[162, 163]
Pyrroline-5-carboxylate reductase (P5CR)	Chloroplastic or cytosolic	Western blot analysis	[164]
Proline dehydogenase (PDH)	Mitochondrial	Submitochondrial fractionation	[165, 166]
Ornithine-a-aminotransferase (a-OAT)	Mitochondrial	PCR, southern analysis, northern analysis	[72]
Arginase (ARG)	Mitochondrial	Cosedmentation and radioactivity	[167]
Ornithine carbamoyl transferase (OCT)	Mitochondrial	Gel sieving and anion exchange method	[168]
Pyrroline-5-carboxylate dehydrogenase (P5CDH)	Mitochondrial; cytosolic	Submitochondrial fractionation	[165]

**Table 3:** Elicitors of the proline biosynthesis pathway in plants.

**Species**	**Elicitors**	**Activity**	**References**
*Vicia faba*	Ascorbic acid	Stimulation of the pentose phosphate pathway	[169]
*Panicum miliaceum* L.	Selenium/zinc	Accumulation of more proline and better growth	[16, 67, 170]
*Setaria italica* L.	Zinc	Accumulation of more proline and better growth	[171]
*Rubia tinctorum* L.	Methyl jasmonate	Increased synthesis from glutamate, which could be originated by GDH from *α*-ketoglutarate	[172]
*Zea mays* L.	Hydrogen peroxide	Increase of activities of P5CS and significant upregulation of P5CS gene expression	[173]
*Oryza sativa* L.	Arsenic oxidearsenous trioxide	**—**	[57]
Rubia tinctorum	Methyl jasmonate	Stimulation of proline synthesis and GDH	[172]
*Brassica juncea* (L.) Czern. et Coss.	Salicylic acid	**—**	[174]
*Arabidopsis thaliana*	Phospholipase C	Phospholipase-based signaling	[175]
*Arabidopsis thaliana*	1-Butanol	**—**	[176]

**Table 4:** Inhibitors of the proline biosynthesis pathway in plants.

**Species**	**Inhibitors**	**Activity**	**References**
*Hordeum vulgare* L. var. Prior	ADP	Decreases P5CS activity	[31]
*Beta vulgaris* L.	Carbonylcyanide m-chlorophenylhydrazone	Transport inhibitor	[177]
*Arabidopsis thaliana*	Phospholipase D	**—**	[176]
*Hordeum vulgare* var Larker	l-Thiazolidine-4-carboxylic acid	Inhibits proline dehydrogenase	[178]
*Arabidopsis thaliana*	Egtazic acid (EGTA)	Negative regulation	[176]
*Arabidopsis thaliana*	Verapamil	Negative regulation	[176]

## Data Availability

The authors have nothing to report.
